# Comparative analysis of the genes UL1 through UL7 of the duck enteritis virus and other herpesviruses of the subfamily *Alphaherpesvirinae*

**DOI:** 10.1590/S1415-47572009005000003

**Published:** 2009-01-10

**Authors:** Huixin Li, Shengwang Liu, Zongxi Han, Yuhao Shao, Shuhong Chen, Xiangang Kong

**Affiliations:** Division of Avian Infectious Diseases, National Key Laboratory of Veterinary Biotechnology, Harbin Veterinary Research Institute, Chinese Academy of Agricultural Sciences, HarbinPeople's Republic of China

**Keywords:** duck enteritis virus, UL1-UL7 genes, phylogenetic analysis

## Abstract

The nucleotide sequences of eight open reading frames (ORFs) located at the 5' end of the unique long region of the duck enteritis virus (DEV) Clone-03 strain were determined. The genes identified were designated *UL1*, *UL2*, *UL3*, *UL4*, *UL5*, *UL6* and *UL*7 homologues of the herpes simplex virus 1 (HSV-1). The DEV *UL3.5* located between *UL3* and *UL4* had no homologue in the HSV-1. The arrangement and transcription orientation of the eight genes were collinear with their homologues in the HSV-1. Phylogenetic trees were constructed based on the alignments of the deduced amino acids of eight proteins with their homologues in 12 alpha-herpesviruses. In the UL1, UL3, UL3.5, UL5 and UL7 proteins trees, the branches were more closely related to the genus *Mardivirus*. However, the UL2, UL4, and UL6 proteins phylogenetic trees indicated a large distance from *Mardivirus*, indicating that the DEV evolved differently from other viruses in the subfamily *Alphaherpesvirinae* and formed a single branch within this subfamily.

## Introduction

The duck enteritis virus (DEV), also known as anatid herpesvirus 1 (AnHV-1) or duck plague virus, infects birds of the order *Anseriformes* (ducks, geese and swans) causing a severe epidemic of duck viral enteritis (DVE), also known as duck plague (Kaleta, 1990). This disease is acute, contagious and can result in host death (Davison *et al.*, 1993). Cases of the disease were recorded in domestic ducks in Holland as early as 1923 (Baudet, 1923). In China, the first outbreak of DVE was in 1957 (Huang, 1959). The disease affects waterfowl of all ages. Like other herpesviruses, the DEV may show latency after primary infection and survivors may act as carriers spreading the virus at regular intervals and causing epidemics. Several studies indicated that survivors of DVE may become carriers of the virus for up to four years (Burgess *et al.*, 1979).

DEV is a member of the family *Herpesviridae* with a linear, double stranded genomic DNA. The G+C content of the DEV genome is 64.3%, which is the highest reported for avian herpesviruses of the subfamily *Alphaherpesvirinae* (Gardner *et al.*, 1993). Although some genes have been characterized (Plummer *et al.*, 1998; Hansen *et al.*, 1999; Li *et al.*, 2006; Liu *et al.*, 2007), most of the genomic sequences and organization of the DEV remains unclear. DEV was classified as an unassigned virus of the family *Herpesviridae* by the Eighth International Committee on Taxonomy of Viruses (ICTV) (Fauquet *et al.*, 2005), although it was previously considered a member of the subfamily *Alphaherpesvirinae* (Kaleta, 1990; Shawky and Schat, 2002). The lack of the complete genome sequence and of knowledge on the genomic organization may be one of the reasons that hinders DEV classification.

To elucidate the genomic organization of the DEV, we amplified sequences of unknown regions of the viral genome by ‘targeted gene walking polymerase chain reaction (PCR)'. Herein we present the molecular characteristics of eight genes located at the 5' end of the unique long segment of the DEV genome and compare them with their homologues in other herpesviruses of the subfamily *Alphaherpesvirinae*. These results expand the information on the DEV genome and broaden our understanding of the evolutionary relationships of this virus within the family *Herpesviridae*.

**Figure 1:**
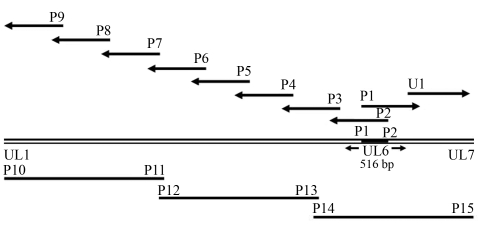
PCR strategy for DEV genome amplification. Primers are indicated and the arrows represent the direction of the ‘targeted gene walking'. The three fragments shown at the bottom were amplified for confirmation of the DEV genomic sequence and each fragment encompasses several smaller overlapped fragments.

**Figure 2:**
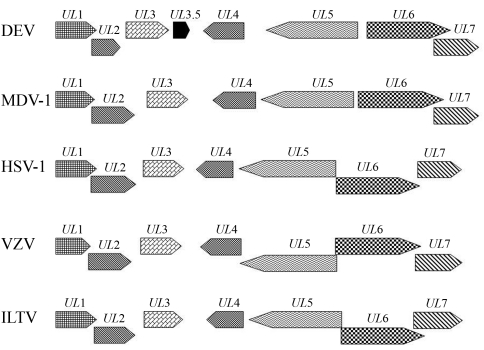
Comparison of the gene content and organization of UL1 through UL7 between the DEV and species of four genera in the subfamily *Alphaherpesvirinae*. The direction of the arrows represent the transcription orientation. Homologous genes of the five herpesviruses were represented with the same pattern.

**Figure 3:**
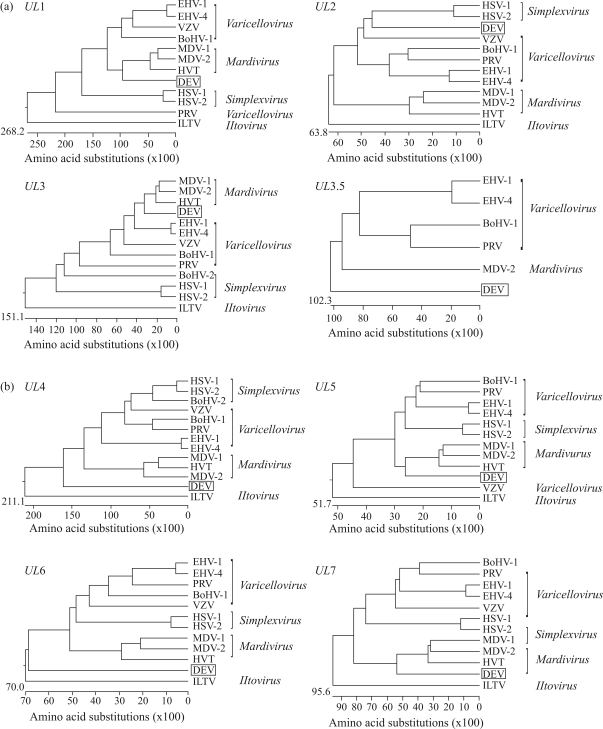
Evolutionary relationships of the eight deduced DEV proteins within the subfamily *Alphaherpesvirinae.* Phylogenetic trees were generated by neighbor-joining. Sequence distances indicated by the scale were calculated using the PAM250 matrix in LASERGENE.

## Material and Methods

### Virus and cells

DEV Clone-03, a commercially available strain of the DEV vaccine, was purified from primary chicken embryo fibroblasts (CEF) using the previously published plaque assay (Li *et al.*, 2006; Liu *et al.*, 2007). The virus was propagated in CEF grown in Dulbecco's Modified Eagle's Medium (DMEM, Gibco-BRL). Viral particles were harvested when the cytopathic effect (CPE) reached 80%. The cell lysate containing DEV was subjected to three freeze-thaw cycles and stored at -70 °C.

### Viral DNA extraction, PCR amplification and sequencing

The cell lysate containing the DEV was centrifugated at 10,000 rpm for 10 min in a F2402H Beckman rotor. Viral DNA was extracted from the supernatant using a Viral DNA Extraction Kit (Invitrogen) according to the manufacturer's instructions. Extracts of a non-infected CEF lysate were prepared as negative controls.

A modified ‘targeted gene walking PCR' (Parker *et al.*, 1991) was used to amplify unknown genes. The PCR strategy used is shown in Figure 1. Primers P1 and P2 were designed according to the sequence of the DEV *UL6* gene (GenBank accession number AF04370). A 516-bp fragment amplified with this set of primers was used as the starting point for amplifying the unknown genes. Four nonspecific walking primers (N1, N2, N3 and N4) were designed based on the conserved region observed after the alignment of the UL6 homologues of herpesviruses. These primers proved useful for ‘walking' the genome of the DEV (Liu *et al.*, 2007). The specific primer P2 and each of the four walking primers were used in the direction of the *UL5* gene to obtain unknown gene fragments. The newly-obtained fragment was sequenced and used as the second starting point for PCR amplification. The same strategy was used to amplify unknown genes in the direction of the UL7 gene with primer P1 and the walking primers. After assembling the gene fragments, three sets of specific primers (P10 and P11, P12 and P13, and P14 and P15) were designed according to the sequences obtained and used to amplify, sequence and confirm the genomic sequences. The primers used in this study are presented in Table 1.

PCR reactions were performed as previously described (Li *et al.*, 2006). The PCR products were analyzed on a 1.0% agarose gel and were sequenced either directly or after cloning into the pMD18-T vector (TaKaRa, Japan). Each region was sequenced at least three times from different PCR products.

### ORF determination

To search for open reading frames (ORFs), the full-length assembled sequence was analyzed using the software Gene Runner (version 3.00, Hasting Software, Inc.). The predicted ORFs were confirmed by tblastx analysis for herpesviruses homologues. Deduced amino acid sequences identified from the DEV ORFs were compared with homologues of alpha-herpesviruses using the MegAlign program (DNAStar, version 7.0).

### Analysis of promoter and polyadenylation signal locations

The assembled sequence of the DEV Clone-03 was submitted to the Berkeley Drosophila Genome project's Neural Network Promoter Prediction, a eukaryotic core promoter search engine (http://www.fruitfly.org/seq_tools/promoter.html). The initial search was performed at a high stringency (cutoff score of 0.85 out of 1.00). The program returned high-scoring core promoters (50-bp-long fragment) along with predicted transcription start sites (TSS). The core promoters found in this search were examined for the presence of TATA box sequences using the TRANSFACFind search engine (http://motif.genome.jp/).

POLYADQ, a eukaryotic polyadenylation (poly A) signal search engine (Cold Spring Harbor Laboratory, http://rulai.cshl.org/tools/polyadq/polyadq_form.html) was used to predict transcription termination signals. All cutoff parameters were initially set at zero to return the location of all AATAAA and ATTAAA consensus signals, along with an associated score between 0 and 1. Signal peptide search (SignalP 3.0) and transmembrane prediction (ConPred II) of the deduced proteins were performed online.

The level of DNA identity between the Kozak's consensus sequence (GCCGCCRCCATGG, R = A/g) (Kozak, 1986) and the flanking 13-nucleotides around the initiator AUG for each ORF was measured.

### Phylogenetic analysis

Alignment was performed by MegAlign in LASERGENE (DNAStar) with the ClustalV method. After a multiple alignment was completed, a neighbor-joining method was employed to reconstruct the phylogeny for the putative alignment of the DEV with 12 alpha-herpesviruses. Reference strains and associated GenBank accession numbers were as follows: Herpes simplex virus 1 (HSV-1), X14112; Herpes simplex virus 2 (HSV-2), NC_001798; Varicella-zoster virus (VZV), NC_001348; Bovine herpesvirus 1 (BoHV-1), NC_001847; Bovine herpesvirus 2 (BoHV-2), D00537; Equid herpesvirus 1 (EHV-1), AY464052; Equid herpesvirus 4 (EHV-4), NC_001844; Pseudorabies virus (PRV), NC_006151; Marek's disease virus type 1 (MDV-1), NC_002229; Marek's disease virus type 2 (MDV-2), NC_002577; Turkey herpesvirus (HVT), AF291866; Infectious laryngotracheitis virus (ILTV), NC_006623.

### Nucleotide sequence accession number

The DNA sequences for the *UL1* through *UL7* genes of the DEV Clone-03 were submitted to the GenBank database and assigned the accession number EF449516.

## Results

### ORF determination, gene arrangement and predicted transcriptional elements

The full-length assembled sequence amplified by ‘targeted gene walking PCR' is 10,374 bp long and includes the *UL1*, *UL2*, *UL3*, *UL3.5*, *UL4*, *UL5*, *UL6* and *UL7* genes. The arrangement of the eight genes and the transcription orientation are shown in Figure 2. The gene *UL3.5*, located between *UL3* and *UL4*, had no homologue in the HSV-1 genome. The *UL1* gene had a 140-nucleotide overlap with the *UL2* gene in the tail to head direction, while the *UL6* gene had a 269-nucleotide overlap with the *UL7* gene in a tail to head orientation.

No poly A sequences were found for the *UL1*, *UL2* and *UL3* genes, but a common poly A signal for these three genes was found immediately downstream of the stop codon of the *UL3.5* gene. Although the *UL1*, *UL2*, *UL3* and *UL3.5* genes shared the same transcription termination site, each gene had its own promoter for transcription initiation. *UL6* and *UL7* shared the same poly A signal sequence located downstream of the *UL7* gene. The promoters, TATA box, poly A signal sequence and the transcription start sites of the eight genes were predicted (Table 2). The Kozak's consensus sequence for each gene was used as a basis for determining the start codon. The context of AUG in *UL1*, *UL3*, *UL5* and *UL7* possessed the feature A (-3) and G (+4), but this feature could not be observed in the AUG context of other four genes.

### Molecular characteristics of the genes *UL1* through *UL7* in the DEV genome

We predicted that the *UL1* gene would encode a protein with 236 amino acids (aa). Amino acid alignment of the DEV UL1 protein with the same protein in 12 alpha-herpesviruses was performed. Various amino acids were conserved, including: Gly_110_, Val_111_, Phe_112_, His_116_, Cys_117_, Glu_121_, Leu_124_, Trp_125_, Ala_132_, Trp_134_, Asn_136_ and Pro_137._ One transmembrane region located from aa 40 through 60 (N-terminus in) of the UL1 protein was predicted. Although the UL1 homologue in the HSV-1 was designated glycoprotein L (gL), no N-linked glycosylation site was predicted in the DEV UL1 protein. Signal peptide analysis showed that the UL1 protein was a signal anchor protein.

The *UL2* gene encoded 157 aa protein and overlapped the 3'-terminus of the *UL1* gene. The DEV UL2 protein was the shortest among the homologues of the 12 reference alpha-herpesviruses. Blast analysis of the amino acid sequence of the UL2 protein showed that it was similar to the uracil-DNA glycosylase of herpesviruses, but no uracil-DNA glycosylase signature consensus was found in the DEV UL2 protein. Eight conserved regions (Figure S1a), mainly located at the C-terminus of the protein, were identified.

UL3 was a conserved protein with 239 aa. Eight casein kinase II phosphorylation sites (Thr_22_, Thr_27_, Ser_51_, Thr_80_, Ser_90_, Thr_94_, Ser_110_, and Ser_111_) were predicted. The nuclear localization signal (NLS) at the C-terminus of the UL3 protein (193-RKPRK-197) was highly conserved among alpha-herpesviruses, in addition to other six conserved regions (Figure S1b).

*UL3.5* encoded a 120 aa protein with a molecular weight of 13.4 kDa. The location of this gene was similar to that of the *UL3.5* gene in the EHV-1, EHV-4, BoHV-1, PRV and MDV-2. Amino acid alignments of the UL3.5 protein with these six virus strains showed weak homology. Blast analyses showed that the DEV *UL3.5* gene was not homologous to a herpesvirus gene. The putative amino acid sequence of the *UL3.5* gene had a higher identity level with the transmembrane 7 superfamily member 3.

The *UL4* gene encoded a 237 aa protein and had little homology with other alpha-herpesviruses homologues (data not shown). Two N-linked glycosylation sites were predicted at amino acids 45 and 223. A 19-aa signal peptide at the N-terminus of the UL4 protein with a cleavage site between the 18th and 19th aa was predicted.

The product of the *UL5* gene in the DEV encoded a 855 aa protein and was highly conserved compared with those in other alpha-herpesviruses. Six conserved helicase motifs were predicted in the UL5 protein. Amino acid alignments showed extensive conserved regions (Figure S1c).

The *UL6* gene encoded a 790 aa protein which shared a high degree of homology with homologues of 11 alpha-herpesviruses. A leucine zipper motif with the consensus sequence L-X_6_-L-X_6_-L-X_6_-L (where X is an arbitrary amino acid) was found in the UL6 protein (463-LESYVNNLFKTIEGLKETNGEL-484), which was consistent with the homologous proteins of other alpha-herpesviruses (Figure S1d). In addition, one transmembrane region containing 21 residues was predicted from aa 606 through 626 (N-terminus out).

The UL7 protein was composed of 321 aa. While no common motifs were shared with the UL7 homologues of 12 alpha-herpesviruses, amino acids 218-LNT-220 and 235-VLP-237 were completely identical among the 12 alpha-herpesviruses. These two sites have unknown functions.

### Phylogenetic analysis of DEV

Phylogenetic trees were constructed based on the alignments of eight DEV proteins with their homologues in 12 alpha-herpesviruses (Figure 3). In all trees, the genera *Simplexvirus*, *Varicellovirus*, *Mardivirus* and *Iltovirus* were distinguished as different clusters. In general, the DEV was most closely related to *Mardivirus*, although it formed a distinct branch. In the UL1, UL3, UL3.5, UL5, and UL7 trees, DEV was closer to *Mardivirus*. The phylogenetic tree of the UL2 protein showed a closer relationship to the cluster of *Simplexvirus*, and the branches of the UL4 and UL6 proteins were very distant from *Mardivirus*.

## Discussion

In this paper we reported partial DNA sequences of the unique long region of the DEV genome containing the *UL1*, *UL2*, *UL3*, *UL3.5*, *UL4*, *UL5*, *UL6* and *UL7* genes. The gene arrangement was collinear to their homologues in the HSV-1, except for the *UL3.5* gene being absent in HSV-1. We analyzed the gene content and gene organization of *UL1* through *UL7* from the typical species of four genera of *Alphaherpesvirinae* (Figure 2). Gene transcription orientation of *UL1* through *UL7* was identical among five herpesviruses. A common feature was the overlapping of *UL1* and *UL2*, and of *UL6* and *UL7.* There were overlaps of the *UL5* and *UL6* genes in HSV-1, VZV and ILTV, but not in MDV-1 and in DEV. In general, gene organization of DEV was more similar to that of MDV-1. To elucidate the taxonomic position of DEV, we performed a comparative analysis of the DEV UL1, UL2, UL3, UL3.5, UL4, UL5, UL6 and UL7 proteins and their counterparts in other virus subfamilies. Our initial analysis was within the family *Herpesviridae*, which showed that DEV was grouped in the subfamily *Alphaherpesvirinae* (data not shown). A second phylogenetic analysis focused on members of the subfamily *Alphaherpesvirinae* was performed in order to further identify the genus of DEV. Phylogenetic trees of the eight proteins of DEV showed a closer relationship to *Mardivirus*, but formed a single branch within the subfamily *Alphaherpesivrinae*.

In general, most HSV genes studied to date were not spliced and were preceded by separate promoter elements including a TATA box. Many HSV ORFs are followed by a poly A signal, but families of HSV genes have also been shown to share one poly A site (McGeogh, 1991). The *UL1*, *UL2* and *UL3* genes of HSV-1 and HSV-2 are each preceded by a TATA box, and there are poly A signals downstream of *UL2* and *UL3* (McGeogh *et al.*, 1988; Worrad and Caradonna, 1988). In the DEV genome, the *UL1*, *UL2*, *UL3* and *UL3.5* genes shared the same poly A signal downstream of *UL3.5*, and the *UL6* and *UL7* genes shared a poly A sequence downstream of *UL7*.

In a eukaryotic cell, the core of the Kozak's consensus is R_-3_CCAUGG_+4_ (R = A/g). R_-3_ is considered the most important flanking residue, followed by G_+4_ (Kozak, 1987). The flanking sequence of AUG in *UL1*, *UL3*, *UL5* and *UL7* possessed the important feature of A_-3_ and G_+4_ that are critical for recognition of AUG by the 40S ribosomal subunit, while the DEV *UL2*, *UL3.5*, *UL4* and *UL6* contexts of AUG did not fit the Kozak rule. We compared the homologous genes in MDV-1 and PRV and not all genes were in good AUG context with Kozak's rule. Although the nucleotides adjacent to AUG (CCRCCAUGG, purine at position -3 and G at position +4) are known to be more important for efficient translation than the rest of the Kozak's consensus, the secondary structure of the RNA can affect the efficiency of translation (Pelletier and Sonenberg, 1987). Further experiments are needed to confirm the true translation start codon.

DEV UL1 did not show significant sequence homology with the HSV-1 UL1 protein, but the gene location and the orientation of *UL1* were equivalent for both viruses. The product of the HSV-1 *UL1* gene is the essential glycoprotein gL (Hutchinson *et al.*, 1992; Roop *et al.*, 1993). Initial analysis of the DEV UL1 protein did not indicate that an N-glycosylation site was present, however further experimental evidence is needed to determine if the UL1 protein is a glycoprotein. HSV-1 gL has been shown to complex with the viral glycoprotein gH and is required for the processing and cell surface expression of gH (Hutchinson *et al.*, 1992). The molecular characteristics of DEV gH have been reported (Li *et al.*, 2006) and additional experiments will be required to determine whether the product of DEV UL1 interacts with gH.

DEV UL2 is a homologue of the HSV-1 UL2 protein, which encodes a non-essential enzyme with uracil-DNA glycosylase activity (Worrad and Caradonna, 1988; Mullaney *et al.*, 1989). The UL2 protein of DEV had no uracil-DNA glycosylase signature sequence. In HSV-1, interruption of the *UL2* ORF by lacZ insertion mutagenesis resulted in a virus which grew normally in tissue culture yet was negative for uracil-DNA glycosylase activity (Mullaney *et al.*, 1989).

DEV UL3 had significant homology with other 12 alpha-herpesviruses. The UL3 protein had eight casein kinase II phosphorylation sites and one NLS (RKPRK) at the C-terminus. We predict that the DEV UL3 protein may be a nuclear phosphoprotein similar to the UL3 protein in HSV-2 (Worrad and Caradonna, 1993). The *UL3* gene is dispensable for viral replication in cell culture in PRV and HSV-1 (Baines and Roizman, 1991; Klupp *et al.*, 2004) which provides a reason for studying the role of the *UL3* gene in DEV.

The deduced amino acid sequence of DEV *UL3.5* had no detectable homology to that of herpesviruses, while it showed high identity with the transmembrane 7 superfamily member 3. Nevertheless, a similar gene location was found for this gene in PRV, BoHV-1, EHV-1, EHV-4 and MDV-2. In PRV, UL3.5 has been shown to be involved in virus egress (Fuchs *et al.*, 1996), but we could not speculate on the function of DEV UL3.5 because of their low homology.

DEV UL4 was predicted to be a glycoprotein with three transmembrane regions. The *UL4* gene in HSV-1 and in PRV is dispensable for virus replication (Fuchs *et al.*, 2006), but the role of the *UL4* gene in the DEV life-cycle remains to be determined.

The DEV UL5 protein contained six conserved helicase motifs which could be found in all members of a superfamily of DNA and RNA helicases. This superfamily contains over 20 members that are present from bacteria to mammalian cells and their viruses. The importance of these motifs has been experimentally addressed in HSV-1 (Zhu and Weller, 1992). Specifically, motif I is directly involved in ATP binding and/or hydrolysis, motif II appears to be required for the coupling of DNA binding to ATP hydrolysis, and residues in motifs III, IV, V, and VI are involved in both DNA binding to ATP hydrolysis and the process of DNA unwinding (Biswas and Weller, 2001). We thus predict that DEV UL5 is a helicase-primase protein.

As a minor capsid protein in HSV-1, UL6 forms the portal for entry of DNA into the capsid (Newcomb *et al.*, 2001). The *UL6* gene of DEV has been used as a target sequence to inhibit virus replication (Mallanna *et al.*, 2006), although the function of its product in the DEV life-cycle is still unknown.

Our homology analysis suggested that the product of the *UL7* gene was not a highly conserved protein in alpha-herpesviruses. The UL7 protein in PRV and in HSV-2 proved to be a structural protein which affected virion formation and virus egress, but the UL7 gene was not considered essential for virus replication (Fuchs *et al.*, 2005).

Despite the large genome of herpesviruses, not all genes are essential for virus replication in cell culture. Until now, the *UL3*, *UL4* and *UL7* genes of HSV-1 and of PRV have been reported to be dispensable for virus replication in cell culture (Baines and Roizman, 1991; Fuchs *et al.*, 2005). Determining if these genes are essential for DEV replication will be important to decide on the use of DEV as a vaccine vector.

## Figures and Tables

**Table 1:** Primers used for PCR amplification.

Primer name	Direction	Primer sequence
P1	Foward	5'-GTGCATGAGGCATTTAGAAC-3'
P2	Reverse	5'-TGCAACGAGGAGAGTTATTG-3'
P3	Reverse	5'-GTTTCATCTAAATACGCTCT-3'
P4	Reverse	5'-TATAAGGGCTGTTTGGAGTG-3'
P5	Reverse	5'-TGCAAAGTACGGTCAAGTGA-3'
P6	Reverse	5'-AGGAGAAACATCCATAGAGT-3'
P7	Reverse	5'-TTTATAACTTACACTCTGGG-3'
P8	Reverse	5'-TCTCTTAGAGGCGTCAATAG-3'
P9	Reverse	5'-TTCCACAAGGAAGTTGCCAG-3'
U1	Foward	5'-CCATCGGATGTACAAAAATG-3'
P10	Foward	5'-GTTGTCGCCGAGGTGTAAAT-3'
P11	Reverse	5'-ACAAGTGATCTGTTCGTGCG-3'
P12	Foward	5'-ACATTACACGGAGGGAGTTT-3'
P13	Reverse	5'-GTCGTGCATCTAACCCCCTA-3'
P14	Foward	5'-ATTTCCATAATAGCCTCTCT-3'
P15	Reverse	5'-TGCAATGAAGATGTAGAAGC-3'
N1		5'-TATAGGTTT(C/A)TGTT-3'
N2		5'-CTTTTGGAGCTG-3'
N3		5'-GAATGTGA(A/g)AA-3'
N4		5'-CATGTCTGCCGA-3'

**Table 2:** Predicted core promoter and polyadenylation elements for the genes UL1 through UL7.

Gene	Length (bp)	Promoter locationa	Promoter score	TATA sequence	TATA location^a^	TSS location	Kozak (of 13)^b^	Poly(A) sequence	Poly(A) location	Poly(A) score	Note
UL1	711	929-978	0.99	CTTTAAA	939-945	969	8/13				The UL1, UL2, UL3, and UL3.5 genes share the same transcription terminal signal
UL2	474	1081-1130	0.91	TATAATA	1089-1095	1121	6/13				
UL3	720	2111-2160	1	TATAAAA	2121-2127	2151	9/13				
UL3.5	363	2702-2751	0.82	TATATAC	2711-2717	2742	6/13	AATAAA	3388-3393	0.322879	
UL4	714	4258-4209^r^	0.91	GACAAAA	4250-4244^r^	4218r	7/13	AATAAA	3431-3426^r^	0.408986	
UL5	2568	7400-7351^r^	0.89	TATAAAC	7390-7384^r^	7360r	7/13	AATAAA	4327-4322^r^	0.280314	
UL6	2373	6665-6714	1	TATTAAT	6673-6679	6705	5/13				The transcripts of the UL6 and UL7 genes are coterminal in the 3' end
UL7	966	9092-9141	1	TATTAAA	9098-9104	9132	5/13	AATAAA	10239-10244	0.319119	

^a^r indicates the 3' to 5' direction. ^b^Identity of the AUG to the Kozak consensus: GCCGCCR_-3_CCAUGG_+4_.

## Supplementary Material

The following online material is available for this article:

Figure S1 Amino acid sequence alignment of the UL2, UL3, UL5, and UL6 genes of the DEV Clone-03 with the corresponding homologues of reference strains in the subfamily *Alphaherpesvirinae.* (a) For UL2, the signature consensus for uracil-DNA glycosylase is underlined and eight conserved regions are boxed; (b) For UL3, six conserved regions are boxed and the nuclear localization signal is underlined; (c) For UL5, six conserved helicase motifs and their locations are presented; (d) For UL6, partial amino acid alignment is shown and the leucine zipper motifs are boxed. Black shades indicate alignments with conservation between 80 and 100% and grey shades represent less than 80% conservation but more than 60% (generated by the software GenDoc).

This material is available as part of the online article from http://www.scielo.br/gmb.
