# Supplementary material for: Comparative analysis of the genes UL1 through UL7 of the duck enteritis virus and other herpesviruses of the subfamily Alphaherpesvirinae
Source: Genet Mol Biol. 2009 Jan 10;32(1):121–8. doi: 10.1590/S1415-47572009005000003 (PMC3032949; doi:10.1590/S1415-47572009005000003)

Figure S1 - Amino acid sequence alignment of the UL2, UL3, UL5, and UL6 genes of the DEV Clone-03 with the corresponding homologues of reference strains in the subfamily *Alphaherpesvirinae.* (a) For UL2, the signature consensus for uracil-DNA glycosylase is underlined and eight conserved regions are boxed; (b) For UL3, six conserved regions are boxed and the nuclear localization signal is underlined; (c) For UL5, six conserved helicase motifs and their locations are presented; (d) For UL6, partial amino acid alignment is shown and the leucine zipper motifs are boxed. Black shades indicate alignments with conservation between 80 and 100% and grey shades represent less than 80% conservation but more than 60% (generated by the software GenDoc).

Figure S1a

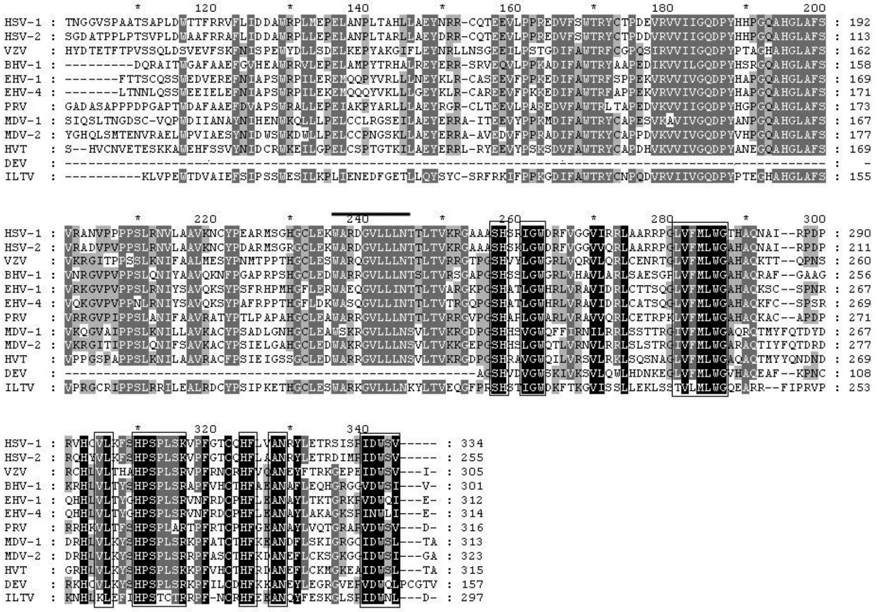

Figure S1b

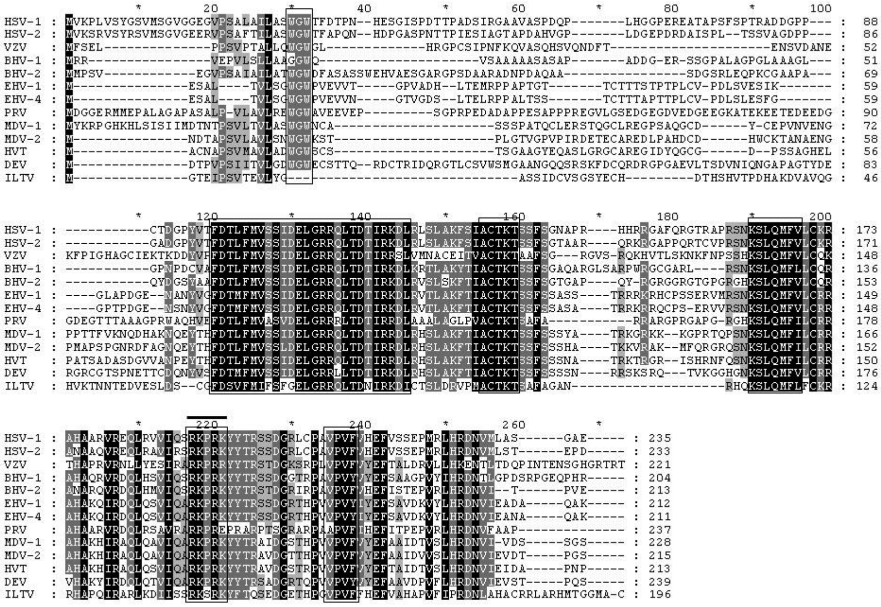

Figure S1c

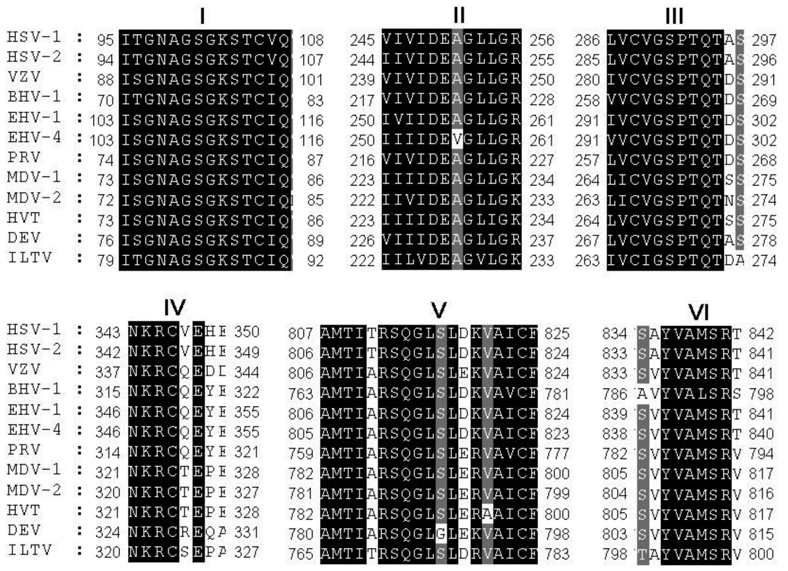

Figure S1d

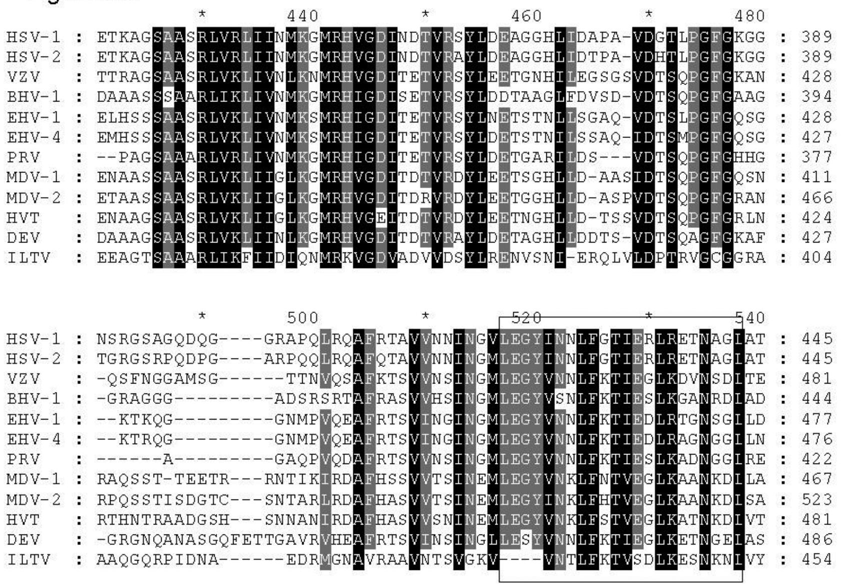

Supplement: Figure S1 — Amino acid sequence alignment of the UL2, UL3, UL5, and UL6 genes of the DEV Clone-03 with the corresponding homologues of reference strains in the subfamily Alphaherpesvirinae. (a) For UL2, the signature consensus for uracil-DNA glycosylase is underlined and eight conserved regions are boxed; (b) For UL3, six conserved regions are boxed and the nuclear localization signal is underlined; (c) For UL5, six conserved helicase motifs and their locations are presented; (d) For UL6, partial amino acid alignment is shown and the leucine zipper motifs are boxed. Black shades indicate alignments with conservation between 80 and 100% and grey shades represent less than 80% conservation but more than 60% (generated by the software GenDoc). [file gmb-32-1-121-suppl1.pdf]
